# Reporting in the abstracts presented at the 5th AfriNEAD (African Network for Evidence-to-Action in Disability) Conference in Ghana

**DOI:** 10.1186/s41073-018-0061-3

**Published:** 2019-01-16

**Authors:** Eric Badu, Paul Okyere, Diane Bell, Naomi Gyamfi, Maxwell Peprah Opoku, Peter Agyei-Baffour, Anthony Kwaku Edusei

**Affiliations:** 1 Department of Health Promotion and Disability Studies, School of Public Health, Kwame Nkrumah University of Science and Technology, Kumasi, Ghana; 2 Business Faculty, Cape Peninsula University of Technology, Cape Town, South Africa; 3 School of Education, University of Tasmania, Hobart, Australia; 4 Department of Health Policy, Management and Economics, Kumasi, Ghana

**Keywords:** Poor reporting, Abstracts, Methodological issues, Disability research, Africa

## Abstract

**Introduction:**

The abstracts of a conference are important for informing the participants about the results that are communicated. However, there is poor reporting in conference abstracts in disability research. This paper aims to assess the reporting in the abstracts presented at the 5th African Network for Evidence-to-Action in Disability (AfriNEAD) Conference in Ghana.

**Methods:**

This descriptive study extracted information from the abstracts presented at the 5th AfriNEAD Conference. Three reviewers independently reviewed all the included abstracts using a predefined data extraction form. Descriptive statistics were used to analyze the extracted information, using Stata version 15.

**Results:**

Of the 76 abstracts assessed, 54 met the inclusion criteria, while 22 were excluded. More than half of all the included abstracts (32/54; 59.26%) were studies conducted in Ghana. Some of the included abstracts did not report on the study design (37/54; 68.5%), the type of analysis performed (30/54; 55.56%), the sampling (27/54; 50%), and the sample size (18/54; 33.33%). Almost all the included abstracts did not report the age distribution and the gender of the participants.

**Conclusion:**

The study findings confirm that there is poor reporting of methods and findings in conference abstracts. Future conference organizers should critically examine abstracts to ensure that these issues are adequately addressed, so that findings are effectively communicated to participants.

**Electronic supplementary material:**

The online version of this article (10.1186/s41073-018-0061-3) contains supplementary material, which is available to authorized users.

## Introduction

An abstract is a condensed version of a full scientific paper that describes the aim of a study, the methods employed, the results, and the conclusions, including implications for policy and practitioners [1]. The abstract of every article is important to inform the reader about the results that are communicated [2]. In particular, the abstract is relevant as readers often make their preliminary assessment of the study at this stage. In fact, some readers, particularly clinicians, may use information from abstracts to inform their clinical decisions, due to their having limited time and resources [3].

Conversely, some researchers may never publish studies as full journal articles, and so the only published record of a study might be the abstract in the conference proceedings. The abstracts for a conference always yield insights, questions, and interpretations that alter and improve the final manuscript, supposing the authors decide to publish such studies in peer-reviewed journals. In particular, effective abstracts describe the importance of the scientific research performed [1, 4]. The participants in a conference usually make their preliminary assessment of a study using the information presented in the conference abstract. However, abstracts presented at conferences have largely been criticized as poor [1, 2], particularly in disability research. The poor reporting in conference abstracts may have several implications, particularly communicating incomplete information on findings and conclusions.

Recently, several studies have been undertaken on reporting in abstracts in disability research [5–9]. These studies have largely focused on poor reporting on the methods employed, including sampling, sample size selection, design, and ethical considerations [7, 8, 10]. However, none of these studies have attempted to assess poor reporting in conference abstracts. A literature search that was conducted identified few reviews and commentaries on abstracts, but rather focused on the reporting quality in abstracts in a randomized controlled trial in psychiatry [3], as well as practical lessons for writing conference abstracts [1, 2, 4]. None of these studies have attempted to assess poor reporting in abstracts from a scientific conference on disability.

Consequently, the African Network for Evidence-to-Action in Disability (AfriNEAD), which is a stakeholder group in disability that works to strengthen evidence-based intervention and policies, has organized a series of expert meetings and symposia in different settings in Africa. In previous symposia, the network upgraded the medium into a scientific conference, so as to strengthen collaboration and transform evidence into action. The College of Health Sciences at Kwame Nkrumah University of Science and Technology collaborated with the University of Stellenbosch to host the fifth scientific AfriNEAD conference for 2017 in Ghana.

This study aims to assess incomplete reporting in abstracts presented at the 5th AfriNEAD Conference in Ghana. In particular, the study assesses the content of abstracts in relation to information on the methods used, the results, and the conclusions, as well as how the abstracts meet the standards for reporting in abstracts. The study was facilitated by the following standards for reporting in abstracts: Strengthening the Reporting of Observational studies in Epidemiology (STROBE) Statement—Items to be included when reporting observational studies in a conference abstract [11, 12], as well as previous literature addressing methodological issues in abstracts [13–15].

## Methods

### Eligibility criteria

The study employed a descriptive design to assess the reporting in abstracts presented at the 5th AfriNEAD Conference, held on 7–9 August 2017 in Ghana. The study assessed the content of the abstracts against the standards for reporting [11, 12]. Abstracts included in the study were those that focused on one of the conference sub-themes, namely the following: children and youth with disability; education: early to tertiary; economic empowerment; development process in Africa: poverty, politics, and indigenous knowledge; health and HIV/AIDS; systems of community-based rehabilitation; holistic wellness, sport, recreation, sexuality, and spirituality; and research evidence and utilization, and abstracts of side events. The included abstracts were either structured or unstructured. However, one criterion was that the content of structured and unstructured abstracts should have adequate information that covers the background to the study, the methods used, the results, and the conclusions. Abstracts were also excluded if they were unstructured but did not adequately capture information on the background, the methods, the results, and the conclusions, but merely gave a brief narrative about the study.

### Selection of the included abstracts

**Fig. 1:**
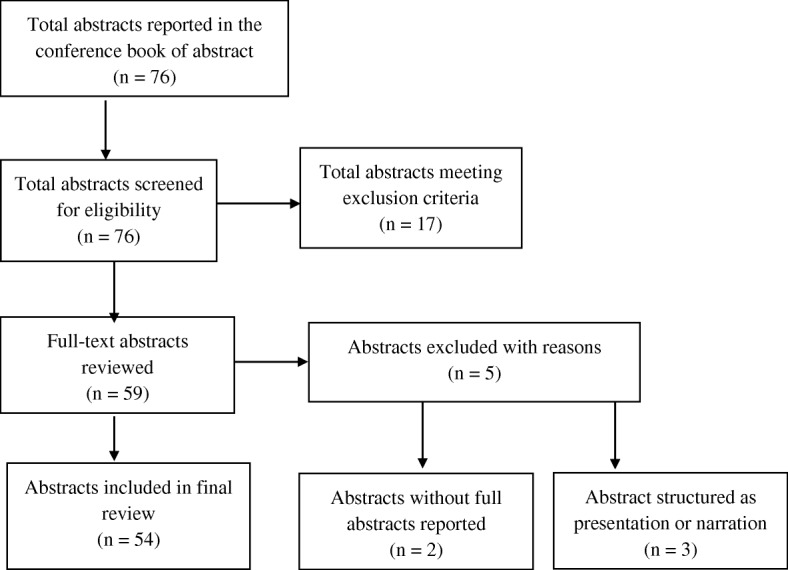
Flow chart of studies included in the review

Three reviewers independently reviewed the titles and the content of the printed conference proceedings, and then approved on those that met the selection criteria. All the conference abstracts that were approved were included in the study. The Preferred Reporting Items for Systematic Reviews and Meta-Analyses (PRISMA) flow chart for systematic reviews [16] was used to illustrate the selection processes (see Fig. 1).

### Data extraction

A data extraction form was developed to extract information from all the included abstracts (see Additional file 1). The data extraction form was developed using the following reporting standards: Strengthening the Reporting of Observational studies in Epidemiology (STROBE) Statement—Items to be included when reporting observational studies in a conference abstract [11, 12], and variables of interest that have been captured in previous literature [13–15]. The data extraction form was divided into subsections, and it covered information on the background of the authors, the sub-themes, the objective of the study, the methodological issues, and the results. Three reviewers were involved in the extraction of data from all the included abstracts.

### Data synthesis

Descriptive statistics, including frequencies, means, standard deviations, and percentages, were used to present the findings. Tables and figures were used to present the results. The analysis was performed using Stata version 15.

## Results

### Description of the abstracts reviewed

The study screened a total of 76 titles of conference abstracts. Of these, 59 met the inclusion criteria, while 17 were excluded. After a review of the full abstracts, a further five were excluded. Overall, 54 abstracts were included in the study (see Fig. 1).

### Characteristics of the included abstracts

More than half of all the included abstracts (32/54; 59.26%) were studies that reported findings from Ghana. About a third of the included abstracts (16/54; 29.6%) focused on the sub-theme “education: early to tertiary,” while more than a tenth each focused on the sub-themes “holistic wellness, sport, recreation, sexuality, and spirituality” (8/54; 14.8%), “children and youth with disability” (7/54; 12.96%), and “health and HIV/AIDs” (7/54; 12.96%). More than two fifths (24/54; 44.44%) of the abstracts targeted people with disabilities, 17/54 (31.48%) used professionals (nurses, doctors, teachers, and stakeholders, including education directors and coordinators), and 5/54 (9.26%) used parents and caregivers (see Table 1).

**Table 1:** Characteristics of included abstracts

Variable	Frequency	Percentage
Participants used in the included studies		
People with disabilities	24	44.44
Professionals (nurses, doctors, teachers, stakeholders)	17	31.48
General students without disability	4	7.4
Caregivers and parents	5	9.26
People without disability	4	7.4
Total	54	100
Sub-themes of abstract		
Children and youth with disabilities	7	12.96
Education: early to tertiary	16	29.63
Economic empowerment	1	1.85
Development process in Africa: poverty	6	11.11
Health and HIV and AIDs	7	12.96
Systems of community-based rehabilitation	4	7.41
Wellness: sports, recreation, sexuality	8	14.81
Research evidence and utilization	5	9.26
Total	54	100
Geographical setting of study		
Africa	3	7.41
Ghana	32	59.26
South Africa	7	12.96
Namibia	2	3.7
Cameroon	2	3.7
Other settings*	8	14.81
Total	54	100

*Kenya, Malawi, Liberia, Nigeria, Tanzania, the USA, and Zimbabwe

### The reporting of methods in the conference abstracts

**Fig. 2:**
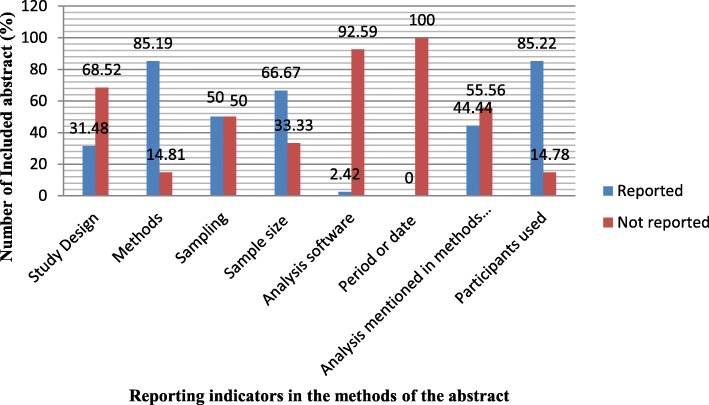
The reporting of methods in the conference abstracts

Two thirds (36/54; 66.67%) of the included abstracts reported sample size in the abstracts, while 18/54 (33.33%) had no information on sample size (see Fig. 2). Most of the included abstracts (37/54; 68.5%) did not report the study design. Of the 17 abstracts that reported the study design, almost half (8/17; 47.06%) used a descriptive design (see Table 2). Most of the abstracts (45/54; 83.33%) reported the methods employed, while 9/54 (16.66%) had no information on the methods employed. Of the abstracts that reported the methods, 35/45 (77.77%) stated that qualitative methods were used (see Table 2).

**Table 2:** Reporting of methods

Variables	Frequency	Percentage
Study design		
Case study	3	17.65
Cross-sectional	1	5.88
Descriptive design	8	47.06
Exploratory design	5	29.41
Total	17	100
Methods		
Qualitative	35	77.77
Quantitative	8	17.77
Mixed methods	2	4.44
Total	45	100
Sampling		
Purposive sampling	18	66.67
Convenience sampling	3	11.11
Snowballing	1	3.70
Simple random and purposive sampling	2	7.40
Simple random sampling	3	11.11
Total	27	
Analysis mentioned in the methods of the abstract		
Thematic content analysis	17	70.84
Descriptive statistics	5	20.83
Both descriptive and thematic analysis	1	4.17
Inferential statistics	1	4.14
Total	24	100

Source: Extracted data, 2017

The study showed that half of the included abstracts (27/54; 50%) did not report the sampling techniques used. Of the abstracts that reported the sampling, 18/27 (66.67%) used purposive sampling (see Table 2). More than half of the abstracts (30/54; 55.56%) did not report the type of analysis performed. However, of the abstracts that reported such information, 17/24 (70.84%) reported thematic analysis.

The majority of the included abstracts (50/54; 92.59%) did not report the analysis software used for the study. Only a few of the abstracts (4/54; 7.41%) reported SPSS as the statistical tool for the analysis. None of the included abstracts reported the date of conducting the study in the abstract.

### The reporting of findings in the conference abstracts

The study extracted information about the results reported in the abstracts (see Table 3). None of the included abstracts reported the age distribution of participants in the abstracts. Similarly, most of the included abstracts (53/54; 98.15%) did not report information about the gender of the participants. Most of the included abstracts (37/54; 68.52%) reported results thematically, while a few (7/54; 12.96%) used descriptive statistics (see Table 3).

**Table 3:** Reporting of findings

Variable	Frequency	Percentage
Age of participants		
Not reported	54	100
Gender of participants		
Reported	1	1.85
Not reported	53	98.15
Type of results reported		
Descriptive statistics	7	12.96
Inferential statistics	1	1.85
Thematic analysis	37	68.52
Both descriptive statistics and thematic analysis	1	1.85
Analysis not clear	8	14.81
Reporting association		
Applicable	6	11.11
Not applicable	48	88.89
Reporting association		
Reported	1	16.67
Not reported	5	83.33
Reporting outcome		
Applicable	43	79.63
Not applicable	11	20.37
Reporting outcome		
Reported	39	90.69
Not reported	4	9.30

Source: Extracted data, 2017

The majority of the included abstracts (48/54; 88.89%) did not report quantitative information that can be used to established associations between the dependent and the independent variables. Of the six included abstracts that were eligible to report such information, only one abstract reported such associations. Most of the included abstracts (43/54; 79.63%) were eligible to report on the primary outcome of the participants. Of the abstracts that were eligible to report on the primary outcome, 39/43 (90.69%) reported on such outcome, while 4/43 (9.30%) did not report on such outcome (see Table 3).

## Discussion

### Strengths and limitations

Our study has some strengths and limitations, which need to be explained. In terms of strengths, the study developed a data extraction form to extract information. Also, the authors followed due process, to ensure that adequate information was gathered and that the information was checked, so as to limit the risk of bias in the reporting of findings (see Table 4). Three reviewers independently reviewed the included abstracts. The reporting of the abstracts confirmed the findings of previous studies on methodological issues in disability research.

**Table 4:** Methods used in the included abstracts

Authors	Title of abstracts	Study design	Methods	Sampling	Data collection	Data analysis
Malonje [17]	*Practicing inclusive early childhood development: an assessment of effectiveness of early childhood development and social intervention for young children with disabilities in Malawi*	Not reported	Literature review	Not applicable	Not reported	Not reported
Efua [18]	*Parental involvement: rethinking the right to education for children with disabilities*	Not reported	Quantitative	Simple random sampling and purposive	Not reported	Not reported
Aboagye et al. [19]	*Caring for children with cerebral palsy: experiences of caregivers at Komfo Anokye Teaching Hospital in Kumasi*	Descriptive design	Qualitative	Simple random sampling	In-depth interviews	Thematic analysis
Taylor et al. [20]	*Disability and leadership: assessing the perceptions of KNUST students towards having disabled persons as leaders*	Cross-sectional	Quantitative and qualitative	Purposive sampling	Semi-structured questionnaire	Not reported
Kyeremateng et al. [21]	*Experiences of caregivers of children with cerebral palsy attending a teaching hospital in Ghana*	Not reported	Qualitative phenomenological	Not reported	In-depth interviews	Thematic analysis
Owusu et al. [22]	*Assessment of level of participation of children with disabilities in extracurricular activities at basic schools in Kumasi Metropolis*	Descriptive design	Qualitative	Purposive sampling	Not reported	Not reported
Maria et al. [23]	*A comparative analysis of objective and subjective inequality between households with and without disabilities in Liberia*	Not reported	Household survey	Not reported	Not reported	Not reported
Sumaila et al. [24]	*An assessment of government support to special schools in the Kumasi Metropolis*	Descriptive design	Qualitative	Purposive sampling	Interviews	Thematic analysis
Awini [25]	*Social interaction patterns between pupils with and without visual impairments in classroom activities in inclusive schools in Ghana*	Not reported	Mixed methods—qualitative and quantitative	Not reported	Questionnaire and focus group discussion	Descriptive statistics and thematic analysis
Ammaru et al. [26]	*Experiences, challenges and coping strategies of teachers in some selected special schools in Ashanti Region*	Descriptive design	Qualitative	Purposive sampling	In-depth interviews	Thematic analysis
Bannieh et al. [27]	*Challenges faced by teachers in teaching deaf learners in selected special schools in Ghana*	Not reported	Qualitative	Purposive sampling	In-depth interviews	Thematic analysis
Owusu-Ansah et al. [28]	*Barriers to inclusive education: the case of Wenchi Senior High School*	Not reported	Qualitative	Purposive sampling	In-depth interviews	Thematic analysis
Baah et al. [29]	*Support services for pupils with low vision in pilot inclusive schools at Ejisu-Juaben Municipality*	Descriptive design	Not reported	Purposive sampling	Likert scale questionnaire	Descriptive statistics
Mariama et al. [30]	*Inclusion of disability studies as a course in the senior high school curricular: perspectives of students at an Islamic and a Secular Schools in the Kumasi Metropolis*	Not reported	Quantitative	Convenience sampling	Structured questionnaire	Descriptive statistics
Wundow et al. [31]	*Perception of teachers on the inclusion of disabled children in inclusive classroom: a case of some selected public basic schools at Sakogu in the Northern Region of Ghana*	Case study	Qualitative	Purposive sampling	In-depth interviews	Not reported
Nseibo [32]	*Experiences of the physically impaired students of Krachi-Nchumbru District of Volta Region of Ghana*	Not reported	Qualitative—phenomenological	Purposive sampling	In-depth interviews	Not reported
Nseibo [33]	*Exploring the experiences of people with mobility impairments in four educational settings in Ghana*	Not reported	Qualitative—interpretive phenomenological	Purposive sampling	In-depth interviews/focus group discussion	Not reported
Mbibeh et al. [34]	*Using assistive technology to enhance inclusive education in the North West Region of Cameroon*	Not reported	Not reported	Not reported	Not reported	Not reported
Chataika and Mutekwa [35]	*Computer skills for every blind child campaign: unlocking educational potential through assistive technology in Zimbabwe*	Not reported	Not reported	Not reported	Not reported	Not reported
Appiah et al. [36]	*Challenges associated with the use of public library services by visually impaired persons in the Kumasi Metropolis*	Descriptive design	Qualitative	Purposive sampling	In-depth interviews/observation	Thematic analysis
Maria [37]	*Integration of rehabilitation and disability concepts/principles into the MBChB undergraduate clinical training*	Case study	Not reported	Not reported	Not reported	Not reported
Mosha and Moshana [38]	*Opportunities and barriers of Moodle the University of Namibia disability community*	Not reported	Qualitative	Not reported	In-depth interviews	Not reported
Yekple and Majisi [39]	*Access to assistive technology for students with visual impairments: the case of University of Education, Winneba in the Central Region of Ghana*	Not reported	Not reported	Purposive sampling	In-depth interviews	Descriptive statistics
Oteng et al. [40]	*Employment of disabled persons in the informal sector: perspectives of physically disabled persons and employers in the Kumasi Metropolis of Ghana*	Exploratory design	Qualitative	Purposive and simple random sampling	In-depth interviews	Thematic analysis
Mile et al. [41]	*Wheelchairs and disability inclusion: the underexploited assistive technology in the North-West of Cameroon*	Not reported	Qualitative	Not reported	Workshop discussion/focus group discussion/observation	Thematic analysis
Kangkoyiri et al. [42]	*The experiences of disabled persons in the Kumasi Metropolis in participating in national elections*	Descriptive design	Qualitative	Simple random sampling	In-depth interviews	Thematic analysis
Owusu et al. [43]	*Increasing access to the criminal justice system for disabled persons in Ghana. The role of assistive technology*	Not reported	Qualitative	Purposive sampling	In-depth interviews	Thematic analysis
Acquah-Gyan et al. [44]	*Challenges of persons with disabilities within the Kumasi Metropolis in accessing information on their human rights*	Exploratory design	Quantitative	Convenience sampling	Structured questionnaire	Descriptive statistics
Peprah et al. [45]	*Challenges of persons with physical disabilities in accessing judicial services in the Kumasi Metropolis*	Not reported	Qualitative—phenomenological enquiry	Convenience sampling	Not reported	Not reported
Ohajunwa et al. [46]	*An Africa centered perspective on assistive technology: informing sustainable outcomes*	Not reported	Not reported	Not reported	Not reported	Not reported
Devlieger [47]	*Urine incontinence, the catheter, and the challenges of African advocacy*	Not reported	Review	Not reported	Not reported	Not reported
Nartey et al. [48]	*Barriers to health care for people living with disability in a teaching hospital, Ghana: the case of the deaf*	Not reported	Qualitative	Purposive sampling	Not reported	Grounded theory
Dadzie et al. [49]	*Knowledge and usage of assistive devices among persons with disabilities in the Kumasi Metropolis*	Not reported	Quantitative	Purposive sampling	Structured questionnaire	Descriptive statistics
Kaundjua [50]	*Health information and health care services among the deaf community in Namibia*	Not reported	Qualitative	Purposive sampling	In-depth interviews/focus group discussion	Not reported
Nadutey et al. [51]	*Menstrual hygiene management: knowledge and practices among female adolescent with disability in Kumasi*	Exploratory design	Qualitative	Purposive sampling	In-depth interviews/focus group discussion	Thematic analysis
Bakari et al. [52]	*Knowledge on and barriers to family planning services by the deaf in the Kumasi Metro*	Descriptive design	Quantitative	Simple random sampling	Questionnaire	Descriptive statistics
Oppong et al. [53]	*Mental health registry in Kumasi: epidemiology of cases reporting to the Hospital*	Not reported	Not reported	Not reported	Electronic online database (questionnaire)	Not reported
Strachan [54]	*A different way of seeing – using assistive technology to live a fully productive life*	Not reported	Qualitative (demonstration and storytelling)	Not reported	Not reported	Not reported
Forkuor et al. [55]	*Caring for the intellectually disabled: motivations, challenges and coping strategies*	Not reported	Qualitative	Not reported	Semi-structured interviews and observation	Not reported
Ned and Ndzwayiba [56]	*The complexity of disability inclusion in the workplace: a south African study*	Case study	Not reported	Not reported	Not reported	Not reported
Oderud [57]	*I hear you – a new hearing concept for low income settings*	Not reported	Participatory approach (qualitative)	Not reported	Not reported	Not reported
Boot et al. [58]	*Improve access to assistive technology for people with intellectual disabilities globally*	Not reported	Qualitative—systematic review	Not reported	Not reported	Not reported
Omoniyi et al. [59]	*Exercise for individuals living with disability: the unwelcome reality*	Not reported	Review	Not reported	Not reported	Not reported
Bukhala [60]	*Sports equipment and technology in developing nations: grassroots initiatives to enhance parasports in Kenya*	Not reported	Systematic review	Not reported	Not reported	Not reported
Ampratum et al. [61]	*Views of Christian religious leaders on the involvement of persons with disabilities in church activities*	Exploratory design	Qualitative	Purposive sampling	In-depth interviews	Thematic analysis
Acheampong et al. [62]	*Maltreatment in marriage; the silent killer, experiences of disabled persons in Yendi Municipality of Ghana*	Exploratory design	Qualitative	Snowballing	In-depth interviews	Thematic analysis
Braathen et al. [63]	*Disability, sexuality and gender: stories from South Africa*	Not reported	Qualitative—participatory	Not reported	In-depth interviews	Not reported
Carew et al. [64]	*Understanding negative attitudes toward the sexual rights and sexual health care access of people with physical disabilities in South Africa*	Not reported	Survey (quantitative)	Not reported	Not reported	Not reported
Haruna [65]	*Assistive technology on female gender in Nigeria: issues and challenges*	Not reported	Content analysis—secondary data analysis	Not reported	Not reported	Not reported
Matter et al. [66]	*AT-INFO-MAP*	Not reported	Document review	Not reported	Not reported	Not reported
Kelley and Harniss [67]	*Assistive technology act programs in the United States*	Not reported	Not reported	Not reported	Not reported	Not reported
Lynn et al. [68]	*Using assistive technology to improve communication, knowledge, and skills in communities of practice and disability inclusive development*	Not reported	Qualitative— narrative synthesis	Not reported	Not reported	Thematic analysis
Osabutey and Osabutey [69]	*The dermatoglyphic patterns of students in special schools compared to those in normal public schools*	Not reported	Quantitative	Not reported	Electronic data collection	Descriptive and inferential statistics
Mduzana et al. [70]	*Suitability of the tool; guidelines for screening of prosthetic candidates: lower limb; for use in Eastern Cape Province*	Not reported	Qualitative	Not reported	Focus group discussion	Not reported

Source: Included abstracts

Our study has several limitations, however, which are mostly associated with the scope and type of the included abstracts. The study was limited to abstracts from one AfriNEAD conference. This suggests that the sample size is too small to make inferences about disability research in general. Limiting abstracts to one AfriNEAD conference may limit access to similar incomplete reporting in past AfriNEAD symposia.

### The reporting of methods and results in the conference abstracts

In the current study, 68.5% of the included abstracts lacked information on the study design, while 14.8% did not report the type of data. This finding implies that there is poor reporting of methodological information, namely study design and type of data used. The incomplete reporting in abstracts implies that readers may have difficulty understanding how the study was conceptualized, as well as the type of data that was used to achieve the results. In particular, reporting study design and methods in conference abstracts is important to inform readers about the broader picture of the study, including the mix of data that is required to achieve the study objective [2]. Omission of such information at the abstract level may create uncertainty among readers. Poor reporting of methods means that readers cannot make concrete and firm conclusions about the subject. This finding can inform future conference organizers on effective ways to address methodological issues. In particular, future scientific abstracts should adequately highlight the relevant methodological issues, such as study design and methods to effectively communicate the findings [2].

The study highlighted that more than half of the included abstracts reported the sample size, while a few did not report such information. Reporting sample size in the abstract is relevant to provide evidence about the participants. Reporting sample size further enables the reader to better understand the representativeness and generalizability of the findings. Although most of the included abstracts reported the sample size, the 33.3% that lacked information on the sample size could provide misleading information to readers. This implies that readers may not be adequately informed about the findings presented in the abstracts. The few abstracts that lacked information on sample size demonstrate poor reporting. This finding confirms the findings of earlier studies on incomplete reporting [1, 2, 4]. Conference abstracts, particularly in disability research, should therefore adequately report the sampling approaches used, so as to inform readers. Scientific committees of conferences, particularly in disability research, should ensure that the sample size of participants is captured in the abstracts, to effectively communicate the findings.

In addition, reporting of the sampling technique used in abstracts is relevant to inform readers about the representativeness of participants, so as to avoid bias. However, about 50% of the included abstracts did not report on the sampling technique. Lack of information on sampling technique in the abstract implies that readers may not be able to generalize the findings reported in the abstract. This finding confirms earlier incomplete reporting in disability research [7, 8, 10]. In particular, the poor reporting in conference abstracts in previous disability research is mostly associated with poor sampling. Our finding demonstrates that conference abstracts should aim to report information on the sampling approach, in order to help readers understand the process involved in selecting participants.

Furthermore, the current study highlighted that 55.56% of the included abstracts did not report the type of analysis performed (whether descriptive or inferential statistics or a qualitative analysis approach). Similarly, some background characteristics, namely age distribution and the gender of participants, were not reported in the abstracts. This finding demonstrates that there is incomplete reporting of results in the abstracts. The results section of the conference abstract appears to be the most significant section that addresses the background characteristics of participants and the primary and secondary outcomes [2]. However, the poor reporting of findings indicates that conference participants will not be adequately informed about the research question and therefore will be unable to explore outcomes, associations, or risk factors. This finding demonstrates that conference abstracts should ensure that the results section includes all relevant information, including age and gender of participants. The poor reporting of results in conference abstracts confirms the findings of earlier studies in disability research [7, 8, 10]. The poor reporting in disability research has largely pertained to incomplete reporting of findings. In some instances, incomplete reporting is largely recorded in full papers, rather than in abstracts.

## Conclusion

The study aims to assess the reporting in the abstracts presented at the 5th African Network for Evidence-to-Action in Disability (AfriNEAD) Conference in Ghana. Our findings confirm that there is poor reporting of methods and findings in conference abstracts. Poor reporting is associated with lack of information about the study design, the methods used, the sampling, the sample size, and the type of analysis performed. Our findings established that reporting evidence in conference abstracts should adequately address all relevant issues. In particular, future conferences on disability research should aim to address the study design, the type of data included, the sampling, the sample size, and the type of analysis employed.

Conference organizers should critically examine abstracts to ensure that these methodological issues are adequately addressed, so that findings are effectively communicated to the participants. The call for abstracts should clearly elaborate the reporting standards, particularly the required content in terms of objectives, methods, results, and conclusions, as well as practical implications for policy and practice. This can help to avoid any incomplete reporting of information in conference abstracts.

## Additional file

Additional file 1: Data extraction form [11, 12]. (DOCX 18 kb)

## Abbreviations

AfriNEAD: African Network for Evidence-to-Action in Disability
ENTREQ: Enhancing Transparency in Reporting the Synthesis of Qualitative Research
GRIPP2: Guidance for Reporting Involvement of Patients and the Public
SD: Standard deviation
STROBE: Strengthening the Reporting of Observational studies in Epidemiology
