# Supplementary material for: Reporting in the abstracts presented at the 5th AfriNEAD (African Network for Evidence-to-Action in Disability) Conference in Ghana
Source: Res Integr Peer Rev. 2019 Jan 16;4:1. doi: 10.1186/s41073-018-0061-3 (PMC6335780; doi:10.1186/s41073-018-0061-3)
Supplement: Supplementary file 1 — Data extraction form [11, 12]. (DOCX 18 kb) [file 41073_2018_61_MOESM1_ESM.docx]

***Additional file 1 Data extraction form*** ([11](#_ENREF_11), [12](#_ENREF_12))

| Study number in the book of abstract |  |
| --- | --- |
| Study ID |  |
| Title of paper | |
|  | |
| Author(s) |  |
| Lead author contact details |  |
| Funder/sponsoring organization |  |
| Geographical Coverage   - Country/ Region |  |
| **Sector**  A report may focus on one or more of the following sub-themes:   - *Children and youth with disabilities* - *Education: early to tertiary* - *Economic empowerment* - *Development process in Africa: poverty, politics and indigenous knowledge* - *Health and HIV & AIDs* - *Systems of Community-Based Rehabilitation* - *Wellness: Sports, Recreation, Sexuality and Spirituality* - *Research evidence and Utilization* - *Abstracts of side events* |  |
| **Reporting standard** |  |
| **Please state the aim of the study** | |
|  | |
| ***Methodology (If applicable)*** |  |
| Study design |  |
| Data collection approaches/type of study |  |
| Participants of included studies  Disability/non-disabled   - If disability, please specify type |  |
| No. of participants/included studies |  |
| Location/setting |  |
| Participant sampling |  |
| Type of analysis performed |  |
| Software used to analyse data |  |
| Recruitment dates |  |
| ***Results (If applicable)*** |  |
| No of participants *ie sampled against response rate;* |  |
| Age range |  |
| Gender |  |
| Reporting findings   - *Please specify type of results reported? ie descriptive statistics, inferential statistics, thematic content analysis* |  |
| Describe statistical methods |  |
| *For quantitative data….*   - *Please specify the type of association reported? ie including those used to control for confounding* |  |
| *For qualitative data….* |  |
| - *Please specify if* ***themes/finding*** *were presented?* |  |
| - *Please specify if the* ***outcomes*** *of participants were presented?* |  |
| *Conclusion and implications* |  |
| - *Please specify if the conclusion is adequately presented? If no, give reasons* |  |
| Recommendations and other information to be considered | |
|  | |
